# Primary Tumor Fluorine‐18 Fluorodeoxydglucose (^18^F‐FDG) Is Associated With Cancer-Associated Weight Loss in Non-Small Cell Lung Cancer (NSCLC) and Portends Worse Survival

**DOI:** 10.3389/fonc.2022.900712

**Published:** 2022-06-24

**Authors:** Santiago Olaechea, Bhavani S. Gannavarapu, Christian Alvarez, Anne Gilmore, Brandon Sarver, Donglu Xie, Rodney Infante, Puneeth Iyengar

**Affiliations:** ^1^Center for Human Nutrition, University of Texas Southwestern Medical Center, Dallas, TX, United States; ^2^Department of Radiation Oncology, University of Texas Southwestern Medical Center, Dallas, TX, United States; ^3^McGovern Medical School, University of Texas Health Science Center, Houston, TX, United States; ^4^Academic Information Systems, University of Texas Southwestern Medical Center, Dallas, TX, United States

**Keywords:** cachexia, positron- emission-tomography, lung neoplams, palliative cancer care, Warburg effect, lipid mobilization, sarcopenia, weight loss

## Abstract

**Aim:**

To investigate the diagnostic potential of and associations between tumor ^18^F‐FDG uptake on PET imaging and cancer-associated weight loss.

**Methods:**

774 non-small cell lung cancer (NSCLC) patients with pre-treatment PET evaluated between 2006 and 2014 were identified. Using the international validated definition of cachexia, the presence of clinically significant pretreatment cancer-associated weight loss (WL) was retrospectively determined. Maximum Standardized Uptake Value (SUV_Max_) of ^18^F‐FDG was recorded and dichotomized based on 3 experimental cutpoints for survival analyses. Each SUV_Max_ cutpoint prioritized either survival differences, total cohort comparison sample sizes, or sample size by stage. Patient outcomes and associations between SUV_Max_ and cancer-associated weight loss were assessed by multivariate, categorical, and survival analyses.

**Results:**

Patients were found to have an increased likelihood of having WL at diagnosis associated with increasing primary tumor SUV_Max_ after controlling for potentially confounding patient and tumor characteristics on multivariate logistic regression (OR 1.038; 95% CI: 1.012, 1.064; *P=0.0037*). After stratifying the cohort by WL and dichotomized SUV_Max_, both factors were found to be relevant in predicting survival outcomes when the alternative variable was constant. Of note, the most striking survival differences contributed by WL status occurred in high SUV_Max_ groups, where the presence of WL predicted a median survival time detriment of up to 10 months, significant regardless of cutpoint determination method applied to categorize high SUV_Max_ patients. SUV_Max_ classification was found to be most consistently relevant in both WL and no WL groups.

**Conclusions:**

The significant positive association between significant pretreatment cancer-associated weight loss and primary tumor SUV_Max_ underscores increased glucose uptake as a component of catabolic tumor phenotypes. This substantiates ^18^F‐FDG PET analysis as a prospective tool for assessment of cancer-associated weight loss and corresponding survival outcomes. Furthermore, the survival differences observed between WL groups across multiple SUV_Max_ classifications supports the importance of weight loss monitoring in oncologic workups. Weight loss in the setting of NSCLCs with higher metabolic activity as determined by ^18^F‐FDG PET signal should encourage more aggressive and earlier palliative care interventions.

## 1 Introduction

Cancer cachexia is a multifactorial syndrome characterized by skeletal muscle wasting and depletion of adipose stores (1, 2). There are limited tools beyond overt weight loss documentation routinely utilized in the diagnosis and risk assessment of cachexia manifestation (3). Evolving preclinical research has identified various explicit mechanisms through which cachexia-inducing tumors manipulate systemic inflammatory and metabolic states ultimately leading to pronounced physical wasting (4–7). This presents a discrepancy between the breadth of mechanistic understanding of cachexia and the limited diagnostic methods employed in clinical practice. The Cachexia Score (CASCO), is an example of a comprehensive method of evaluation that implements these findings, as it incorporates measures of relevant inflammatory markers, metabolic disturbances, physical performance, anorexia, quality of life detriments, and lean body mass changes in addition to weight loss assessment in cachexia grading (8). However, the feasibility of this method is limited by the lack of consistent collection of this data in the clinical setting. Another interfering factor in the development of practical diagnostic tools is the heterogeneity of mechanisms capable of inducing weight loss across diverse tumor histologies and sites. Tumors can generate systemic catabolism through processes including but not limited to: the induction of a metabolically demanding inflammatory state, aggressive depletion of metabolites, and anorexia through disadvantageous hypothalamic signaling or mechanical obstruction from tumor mass effect (4–7, 9–11). As benchside research continues to refine our understanding of cancer cachexia at the molecular and cellular level, we set out to probe for diagnostic potential in routinely obtained tumor imaging characteristics.

Positron Emission Tomography (PET) is a functional imaging technique commonly used in the initial staging of patients with newly diagnosed solid tumors. PET is often performed with the intravenous administration of fluorine-18-fluoro-2-deoxy-D-glucose (^18^F‐FDG), a positron emitting glucose molecule. ^18^F‐FDG accumulates in organs with high glucose utilization such as the brain, heart, and liver. Malignant cells demonstrate increased uptake of ^18^F-FDG on PET due to overexpression of GLUT receptors and elevated intracellular levels of hexokinase, which generate increased rates of glycolysis (6, 12, 13). Another contributing phenomenon is the Warburg effect, in which tumor cells preferentially undergo anaerobic glycolysis to produce ATP, regardless of being in nonhypoxic conditions (14). The endpoint of these metabolic processes is fluorinated glucose trapped within the cell with characteristic increased activity on PET. The measure of ^18^F-FDG uptake is commonly quantified using standardized uptake values (SUV), a measurement of tracer uptake in a lesion normalized to injected activity and volume of distribution (15, 16). SUV should therefore function as a measure of tumor glycolytic capacity. This might be particularly relevant within the context of cancer-associated weight loss and cachexia, as escalated glucose consumption by tumors can distort systemic metabolic flux and suggest increasingly malignant genetic dysregulation through mechanisms such as the Warburg effect. Our research team previously demonstrated the relevance of ^18^F‐FDG uptake in incidence of significant pretreatment weight loss and survival for patients with gastroesophageal cancer (17). Prior studies have supported alternate uses of PET imaging for the detection of cancer cachexia (18), linking low hepatic uptake (19) and increased metabolically active brown adipose tissue uptake (20) of ^18^F‐FDG with the syndrome.

The clinical hallmark of cancer cachexia is unintentional weight loss. The presence of this finding at the time of detection of non-small cell lung cancer (NSCLC) portends poor survival, even after controlling for other factors such as age, sex, comorbidity, and tumor stage (21–23). Although targeted interventions are currently limited (24–26), evidence highlighting the benefits of nutritional rehabilitation for weight-losing cancer patients continues to emerge (27–30). Therefore, early identification of patients bearing tumors with metabolic ramifications is essential. The purpose of this study was to determine the association between primary tumor ^18^F‐FDG uptake *via* Maximum SUV (SUV_Max_) and the presence of significant cancer-associated weight loss at the time of diagnosis, prior to any cancer-directed intervention, in a large cohort of NSCLC patients. We also aimed to investigate differential survival outcomes based on SUV_Max_ and pretreatment weight loss. Further characterization of the effects of and potential associations between these factors may clarify metabolic disruptions exerted by tumors that lead to weight loss. Moreover, evaluating the prospective utility of weight assessment and tumor ^18^F‐FDG uptake might help providers identify NSCLC patients with poor prognoses.

## 2 Methods

### 2.1 Population Cohort

Using a prospectively maintained tumor registry from a single tertiary care center and retrospective patient electronic medical record review, we identified 774 patients with NSCLC diagnosed between 1/1/2006 and 12/31/2013 with pre-treatment PET available for review. Exclusion criteria for this study included synchronous or metachronous malignancy and incomplete data. Patients with carcinoma in situ, carcinoid, neuroendocrine, lymphoma, melanoma, or sarcoma tumor histologies were excluded. Patient and tumor characteristics, including age, sex, race, date of death/last follow up, Charlson comorbidity index (CCI) score, primary tumor site, tumor grade, and cancer stage, were recorded. Descriptive statistics were used to summarize patient and tumor characteristics. The 7^th^ and 8^th^ editions of the American Joint Committee on Cancer (AJCC) staging system were adopted in 2009 and 2018, respectively, but patient stage was not adjusted for the purpose of this study. This study was approved by our Institutional Review Board at UT Southwestern.

### 2.2 Assessment of Cancer-Associated Weight Loss

We reviewed medical records—including vital signs, physician notes, and dietician notes at the time of cancer diagnosis but before any therapeutic measure—for documented weight loss and associated symptoms. At our health system, patients are routinely weighed as a part of each office visit, and this measurement is documented in the electronic medical record. Patients missing weight data before the initiation of therapy were excluded. Determination of clinically significant pretreatment cancer-associated weight loss (WL) was based on the validated international consensus definition of cancer cachexia (1). WL was defined as unintentional weight loss > 5% within 6 months preceding cancer diagnosis in patients with body mass index ≥ 20 kg/m^2^ or unintentional weight loss > 2% in patients with body mass index < 20 kg/m^2^. Patients with stable weight, weight gain, or purposeful weight loss were classified as not having WL. When multiple measures of weight were available in the pre-treatment period, a consistent weight decrease was required for a patient to be classified as having WL.

### 2.3 Positron Emission Tomography (PET) and SUV_Max_ Cutpoint Determination

PET with ^18^F-FDG was obtained in all analyzed patients at the time of diagnosis and prior to any oncologic intervention. After a standard uptake phase of 60-90 minutes, each patient underwent computed tomography (CT) imaging from the level of the midbrain to the midthigh at 3 mm intervals with arms raised above the head. Following completion of the CT portion of the study, 3D emission images were obtained through the same distance. Oral contrast was used in all patients, unless otherwise contraindicated. The CT images were generated for the purpose of PET image optimization and for anatomical correlation of PET findings. Each PET report was generated by a trained nuclear medicine physician. SUV_Max_ of the primary tumor, serum blood glucose level at the time of scan, and time interval between ^18^F‐FDG injection and scan initiation, when available, were recorded.

We created 3 experimental SUV_Max_ cutpoints. A cutpoint optimizing survival was calculated through the method of Contal and O’Quigley (31, 32), which determined the optimal primary tumor SUV_Max_ threshold for survival time. The total cohort median cutpoint prioritized sample size by dichotomizing the cohort using the overall median SUV_Max_. The stage medians cutpoint method similarly prioritized sample size and stratified the patients into high/low SUV_Max_ groups by their respective stage median SUV_Max_.

Tumor PET images and L3 CT slices from the time of cancer diagnosis were obtained for representative patients from relevant groups. PET images were obtained to demonstrate tumor ^18^F-FDG uptake. The L3 level was selected from CT imaging for visualization of psoas and erector spinae muscle groups. Both representative patients had matching gender, tumor histology, and tumor stage at diagnosis.

### 2.4 Statistics

All tests were two-sided and performed at the 5% significance level. SPSS Statistics version 28.0.1.1(International Business Machines, Armonk, NY).

#### 2.4.1 Multivariate

Multivariate logistic regression (N=700) was conducted with WL at diagnosis as the dependent binary variable. Covariates included in this analysis were patient sex, age at diagnosis, race, alcohol history, tobacco history, and CCI score, as well as tumor characteristics including histology, stage and SUV_Max_. All variables were categorical with the exception of age at diagnosis and tumor SUV_Max_. Due to tumor grade limiting the sample size available for analysis, the parameter was not included in the primary multivariate analysis. The results from the limited sample (N=408) multivariate analysis including tumor grade as a covariate are included in **Supplementary Table 1**.

#### 2.4.2 Categorical

Chi square testing was carried out to evaluate differences between expected and observed incidence of WL in high and low SUV_Max_ groups. Tests were repeated for each cutpoint method.

#### 2.4.3 Survival

Survival probability was plotted using Kaplan-Meier estimator. Event occurrence was defined by patient death. Time to censoring or event was provided by the time in months from the cancer diagnosis date to the date of patient death or last contact, respectively. Significance of survival differences were evaluated through log-rank testing.

Groups evaluated for survival included the overall cohort, patients with or without WL at diagnosis, and patients with high or low SUV_Max_. Further analyses divided the cohort 4 groups on the basis of +/- WL at diagnosis and high/low SUV_Max_, in which log-rank testing was done across groups with one differing variable. All tests involving SUV_Max_ were repeated for each cutpoint method

## 3 Results

### 3.1 Patient Cohort

Patient characteristics summarized in **Table 1**. The median age at diagnosis was 66 years (interquartile range, 58-74); 47.90% of patients were female and 34.20% were non-white. 27.80%, 10.80%, 32.30%, and 29.10% patients had stage 1, 2, 3, and 4 NSCLC, respectively. WL was identified in 24.70% of patients.

**Table 1:** Patient Characteristics and Incidence of Significant Pretreatment Cancer-Associated Weight Loss (WL).

Characteristic	Patient Count
**Age at Diagnosis, Median (IQR)**	66 (58, 74)
**Female Sex, (%)**	371 (47.90%)
**Race, N (%)**	
White	496 (65.80%)
Black	194 (25.70%)
Asian or Pacific Islander	23 (3.10%)
Hispanic	41 (5.40%)
**Diabetes, N (%)**	160 (20.70%)
**Tobacco Use History, N (%)**	
Current	265 (34.60%)
Prior	406 (52.90%)
Never	96 (12.50%)
**Alcohol Use History, N (%)**	
Current	334 (46.20%)
Prior	57 (7.90%)
Never	332 (45.90%)
**Histology, N (%)**	
Squamous Cell Carcinoma	213 (27.50%)
Adenocarcinoma	414 (53.50%)
Other or Unknown	147 (19.00%)
**Tumor Grade, N (%)**	
1	33 (7.30%)
2	205 (45.40%)
3	204 (45.10%)
4	10 (2.20%)
**Tumor Stage, N (%)**	
1	214 (27.80%)
2	83 (10.80%)
3	249 (32.30%)
4	224 (29.10%)
**WL Incidence (%)**	191 (24.70%)

### 3.2 Associations Between SUV_Max_ and Cancer-Associated Weight Loss at Diagnosis

After controlling for covariates (sex, age at diagnosis, race, alcohol history, tobacco history, CCI score, histology, stage), SUV_Max_ demonstrated a statistically significant association with the incidence of cancer-associated weight loss at diagnosis with an odds ratio of 1.038 (95% CI: 1.012, 1.064; *P=0.0037*; **Table 2**). The same analysis using the sample-limiting parameter of tumor stage demonstrated an odds ratio of 1.050 (95% CI: 1.011, 1.091; *P=0.0110*; **Supplementary Table 1**).

**Table 2:** Multivariate Logistic Regression Evaluating Relationship of Tumor SUV_Max_ with Patient and Tumor Characteristics as Covariates with Incidence of Significant Pretreatment Cancer-Associated Weight Loss (WL).

Patient or Tumor Factor	Odds Ratio (95% CI)	P-value
**Female Sex**	0.970 (0.662, 1.421)	*0.8741*
**Age at Diagnosis**	1.020 (1.001, 1.040)	***0.0426***
**Race**		
Non-Hispanic Caucasian	Reference	***0.0060***
Black	2.014 (1.327, 3.058)	***0.0010***
Hispanic	2.064 (0.969, 4.396)	*0.0604*
Asian or Pacific Islander	1.162 (0.349, 3.865)	*0.8066*
**Alcohol History**		
None	Reference	*0.0849*
Prior Use	2.069 (1.089, 3.929)	***0.0264***
Current Use	1.153 (0.776, 1.714)	*0.4798*
**Tobacco History**		
None	Reference	*0.2524*
Prior Use	1.116 (0.565, 2.204)	*0.7525*
Current Use	1.525 (0.754, 3.083)	*0.2405*
**Charlson Comorbidity Index**		
0	Reference	*0.2037*
1	0.607 (0.375, 0.982)	***0.0420***
2	0.653 (0.382, 1.117)	*0.1198*
3+	0.764 (0.436, 1.339)	*0.3469*
**Histology**		
Squamous	Reference	*0.4297*
Adenocarcinoma	0.750 (0.478, 1.177)	*0.2111*
Other or Unknown	0.923 (0.546, 1.563)	*0.7667*
**Stage**		
1	Reference	***0.0298***
2	0.732 (0.336, 1.596)	*0.4333*
3	1.543 (0.911, 2.614)	*0.1065*
4	1.850 (1.082, 3.164)	***0.0247***
**Tumor SUV_Max_**	1.038 (1.012, 1.064)	***0.0037***

P-values bolded if significant (P<0.05).

The calculated survival optimizing cutpoint of 6.8 defined 220 patients as low SUV_Max_ and 554 patients as high SUV_Max_. The incidence of WL at diagnosis was 15.00% in the low SUV_Max_ group and 28.52% in the high SUV_Max_ group (*P<0.0001*, **Table 3**).

**Table 3:** Incidence of Significant Pretreatment Cancer-Associated Weight Loss (WL) by High and Low SUV_Max_ Determined by Experimental Cutpoints.

SUV_Max_ Cutpoint Used; Value	WL Incidence	P-value
**Survival-Optimized; 6.80**		***0.0001***
Low SUV_Max_ (N=220)	33 (15.00%)	
High SUV_Max_ (N=554)	158 (28.52%)	
**Total Cohort Median; 10.60**		***<0.0001***
Low SUV_Max_ (N=392)	71 (18.11%)	
High SUV_Max_ (N=382)	120 (31.41%)	
**Stage Medians; 6.70, 9.50, 13.80, 10.95**		***0.0011***
Low SUV_Max_ (N=392)	77 (19.64%)	
High SUV_Max_ (N=382)	114 (29.84%)	
**Overall Cohort (N=774)**	191 (24.70%)	

P-values bolded if significant (P<0.05).

Groups defined by sample size optimizing cutpoints similarly categorized 392 patients into low SUV_Max_ groups and 382 patients into high SUV_Max_ groups. Across groups defined by the total cohort median SUV_Max_ of 10.60, WL incidence in patients was 18.11% and 31.41% in patients with low and high SUV_Max_, respectively (*P<0.0001*). Median SUV_Max_ values for ascending tumor stage values were 6.70, 9.50, 13.80, and 10.95. WL incidence was 19.64% and 29.84% in patients with stage-defined low and high SUV_Max_ values respectively (*P=0.0011*; **Table 3**).

Artifact-corrected PET images at the level of the primary tumor and CT images at the L3 level were obtained for representative patients with and without WL. One patient demonstrated low tumor SUV_Max_ by every cutpoint method and no WL at diagnosis (**Supplementary Figures 1A, B**). The second representative patient had high SUV_Max_ and WL at diagnosis (**Supplementary Figures 1C, D**).

### 3.3 Survival Associations

The median survival for the entire cohort was 24 months (95% CI: 20.222, 27.778). Patients were grouped into one of four groups on the basis of WL status and SUV_Max_ dichotomized above and below the calculated cutpoints. WL and high SUV_Max_ by any cutpoint determination method predicted for poor survival outcomes to a significant degree as demonstrated by log-rank testing (**Supplementary Table 2**).

**Figure 1:**
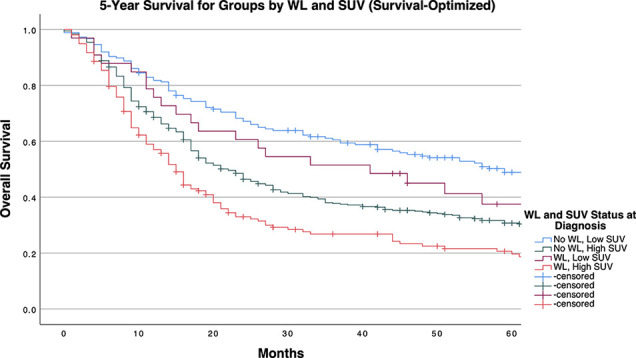
5-Year Overall Survival Stratified by Significant Pretreatment Cancer-Associated Weight Loss (WL) and Primary Tumor SUV_Max_ Group Determined by Survival-Optimized Cutpoint.

**Figure 2:**
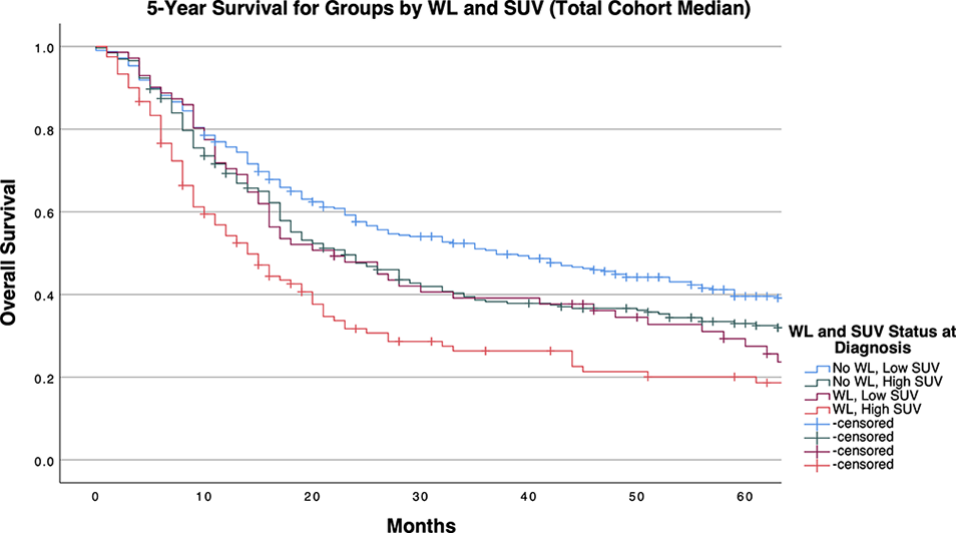
5-Year Overall Survival Stratified by Significant Pretreatment Cancer-Associated Weight Loss (WL) and Primary Tumor SUV_Max_ Group Determined by Cohort Median Cutpoint.

**Figure 3:**
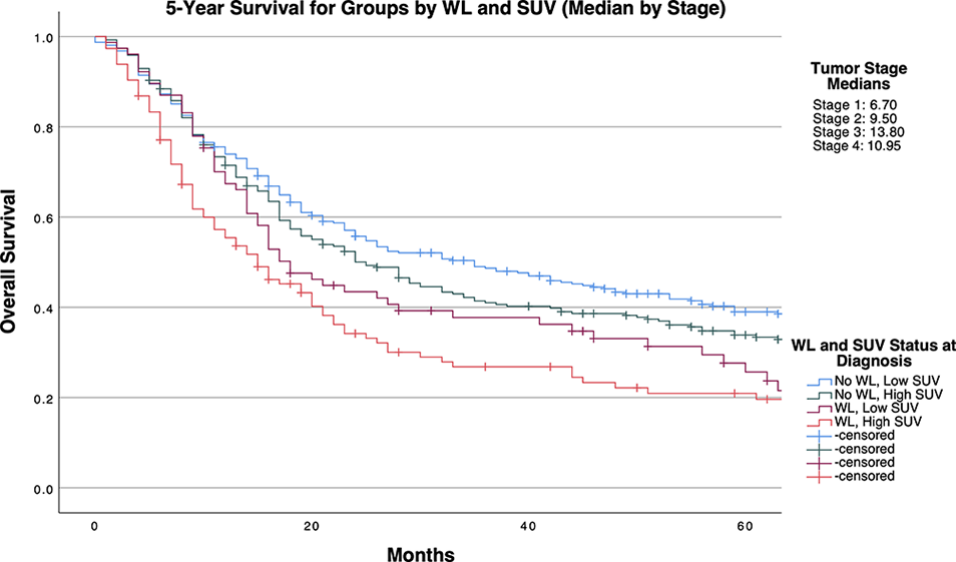
5-Year Overall Survival Stratified by Presence of Significant Pretreatment Cancer-Associated Weight Loss (WL) and Primary Tumor SUV_Max_ Group Determined by Stage-Specific Cutpoints.

**Table 4** and **Figures 1**-**3** demonstrate survival findings and comparisons between groups with varying WL and SUV_Max_ status utilizing the different cutpoint determination methods. Regardless of the cutpoint determining method implemented, both WL and SUV_Max_ status demonstrated significant or near-significant associations with survival when the alternative variable was held constant. Notably, in the stage median cutpoint survival analysis, where sample size matching was prioritized by stage, WL significantly predicted for survival outcome in both low (*P=0.0449*) and high (*P=0.0004*) SUV_Max_ groups.

**Table 4:** 

SUV_Max_ Cutpoint Method	Patient Group	Median Survival Time in Months (95% CI)	Survival Comparison Groups	P-value
**Survival-Optimized**			*WL Constant*	
	No WL, Low SUV	59 (41.548, 76.452)	No WL: High SUV vs Low SUV	***<0.0001***
	No WL, High SUV	22 (18.220, 25.780)	WL: High vs Low SUV	***0.0093***
	WL, Low SUV	41 (14.856, 67.144)	*SUV Constant*	
	WL, High SUV	15 (12.911, 17.089)	Low SUV: No WL vs WL	*0.2342*
	Total	24 (20.222, 27.778)	High SUV: No WL vs WL	***0.0012***
**Total Cohort Median**			*WL Constant*	
	No WL, Low SUV	37 (25.681, 48.319)	No WL: High SUV vs Low SUV	*0.0628*
	No WL, High SUV	23 (17.700, 28.300)	WL: High vs Low SUV	***0.0317***
	WL, Low SUV	22 (11.792, 32.208)	*SUV Constant*	
	WL, High SUV	14 (10.348, 17.652)	Low SUV: No WL vs WL	*0.0813*
	Total	24 (20.222, 27.778)	High SUV: No WL vs WL	***0.0007***
**Stage-Specific Medians**			*WL Constant*	
	No WL, Low SUV	35 (24.243, 45.757)	No WL: High SUV vs Low SUV	*0.3191*
	No WL, High SUV	25 (19.242, 30.758)	WL: High vs Low SUV	*0.1194*
	WL, Low SUV	18 (12.933, 23.067)	*SUV Constant*	
	WL, High SUV	15 (9.659, 20.341)	Low SUV: No WL vs WL	***0.0449***
	Total	24 (20.222, 27.778)	High SUV: No WL vs WL	***0.0004***

P-values bolded if significant (P<0.05).

Broadly, after repeating these analyses in stage-specific subcohorts, SUV_Max_ appeared to retain significance in lower stage tumors, with WL at diagnosis demonstrating associations with survival to a higher frequency in higher stage tumors (**Supplementary Tables 3–5** and **Supplemental Figures 2–4**).

## 4 Discussion

### 4.1 Study Findings

Our study demonstrates a significant positive association between primary tumor SUV_Max_ and pre-treatment cancer-associated weight loss, as well as observable survival detriments related to either and both factors. Through multivariate logistic regression, we accounted for potential confounding factors including patient characteristics (age, sex, race, risk behaviors, comorbidities) as well as conventional tumor classifications (histology, grade, stage), further implicating a mechanistic association between these variables. By dichotomizing SUV_Max_ across multiple cutpoint calculation methods, we provided experimental models for implementing SUV_Max_ diagnostic cutoffs to assess for the presence of cancer-associated weight loss. Regardless of cutpoint method, patients with SUV_Max_ values above a cutpoint had a 1.5 to 2 times increased incidence of pre-treatment cancer-associated weight loss qualifying for diagnosis of cachexia. Mechanistically, these findings underscore the relevance of heightened tumor glucose metabolism to the systemic metabolic derangements classically observed in cachexia. Clinically, our study supports both a heightened consideration of weight loss with increasing tumor SUV_Max_ value and a crucial association between clinically significant pretreatment weight loss identified at diagnosis with survival detriment in patients with both high or low SUV_Max_ tumors.

### 4.2 Clinical Relevance

The associations observed between tumor SUV_Max_ and WL at diagnosis in multivariate analysis suggest a potential prognostic value of PET imaging results in predicting presence or risk of cancer-associated weight loss. By applying multiple cutpoint strategies, we demonstrated that optimal PET measure cutoff values vary depending on the intended prognostic measure. In this project, these measures included WL at diagnosis and survival prediction. Furthermore, our stage stratification of both survival analyses and cutpoint determination indicate differential utility of PET imaging results based on the depth of stratification applied within the cancer type.

To control for the potential bias of later stage tumors (with known survival detriment) being disproportionally classified as having high PET imaging results, our stage-specific median cutpoint group balanced SUV_Max_ groups by stage. Still, the presence of WL at diagnosis significantly predicted a survival detriment independent of SUV_Max_ status, as median survival time was decreased by approximately 17 months in patients with SUV_Max_ below the stage-tailored cutpoint, and 10 months in patients with higher SUV_Max_ values. This trend was consistently observed when analyses were conducted separately for each stage, albeit with diminished significance likely attributable to the limited statistical power permitted by multiple divisions of our cohort. Notably, within this stage stratified cohort, SUV_Max_ surpassed WL as the primarily significant determinant of survival as indicated by the group comparisons in patients with stage 1 tumors. Meeting criteria for WL at diagnosis exceeded SUV_Max_ as the primary determinant factor towards survival detriment. Of note, even when the cutpoint selected favored the relevance of SUV_Max_ in survival (as determined by the method of Contal and O’Quigley), WL significantly predicted a detriment in survival within the high SUV_Max_ group, which retained significance in stage 3 tumors after further stratification (with near-significant results in stage 1 tumors). The relevance of weight loss at presentation to survival of patients with high SUV_Max_ tumors underscores an objective detriment in patient outcomes exerted by cancer-associated weight loss in patients with severe NSCLC tumor phenotypes.

### 4.3 Mechanism of Cancer-Associated Weight Loss and Tumor ^18^F‐FDG Uptake

The observed positive association between primary tumor SUV_Max_ and cancer-associated weight loss is concordant with results observed in murine models of cancer cachexia. In a prior study by Penet et al., *in-vivo* cachexia-inducing MAC16 tumors were characterized by higher ^18^F‐FDG uptake than histologically similar non-cachexia-inducing MAC13 tumors (33). With validation from our large clinical database, these results shed additional light on cancer metabolism, supporting the relevance of elevated tumor glucose consumption (signified by high ^18^FDG uptake) in the induction or presentation of tumor phenotype capable of inducing weight loss.

The study by Penet et al. also found that mice with cachectic MAC16 tumors underwent significant depletion of lipid tissue in their adipose tissue and skeletal muscle, with sparing of lipid at tumor sites (33). These effects highlight the complexity of cachexia manifestation, as there were heterogenous alterations of metabolic state at the systemic level and at the immediate tumor microenvironment. The exact process retains pathophysiologic ambiguity, as current literature supports various mechanisms contributing to the induction of lipolysis in cachexia. These include upregulated inflammatory signaling through IL-6 messengers including leukemia inhibitory factor (34, 35), thermogenic gene induction through tumor signals such as PTHrP (36), and physiologic lipolysis as a downstream metabolic effect of increased energy expenditure through intensified tumor glucose consumption (37). The importance of maladaptive induction of lipolysis in cachexia has been further supported by murine studies demonstrating profoundly elevated activation of thermogenic brown adipose tissue in cachectic over non-cachectic mice, despite matched food intake and thermoneutral conditions (38). In parallel, multiple clinical studies have revealed positive associations between brown adipose tissue induction and cachexia incidence in humans (11, 20, 35, 36). Reasonably, the capability of ^18^F‐FDG PET analysis to measure the activity of brown adipose tissue (39) further augments its potential utility in the assessment and prediction of cancer-associated weight loss and cachexia (18).

### 4.4 Study Limitations

This analysis has a number of limitations. For example, WL was retrospectively assessed from medical records rather than a formal prospective protocol. Although WL was based on the consensus definition of cancer cachexia, more specific diagnostic criteria indicative of a cachectic state - such as specific biomarkers, enhanced lipolysis, and sarcopenia- were omitted due to limitations on what is routinely obtained in patient management (25). Cachexia-specific biomarkers include molecular assays and measures of serum cytokines that were not available for this cohort. The absence of brown adipose tissue analysis on PET reports prohibited our inclusion of maladaptive lipolysis. Regarding sarcopenia, this would have entailed comprehensive evaluations of skeletal muscle mass and function. Although CT imaging permits an estimation of total body skeletal muscle mass through visualization of major muscle groups, we lacked longitudinal imaging data for comparison (40–43). Furthermore, muscle function tests such as grip strength were not routinely obtained for this cohort.

### 4.5 Conclusion

Our study supports a positive association between increased tumor glucose utilization and the development of cancer-associated weight loss that is independent of various potential confounding patient and tumor characteristics. Moreover, we identified a vulnerability of patient survival time to significant weight loss prior to cancer diagnosis, particularly for patients with tumors demonstrating high ^18^F‐FDG uptake.

PET imaging is routinely used for prognosing NSCLC patients using radiolabeled ^18^F‐FDG uptake. Our study validates this function by consistently demonstrating the relevance of SUV_Max_ obtained from this technique in survival prognosis. Given the maladaptive metabolic changes that underpin both cancer-associated weight loss and PET imaging enhancement by ^18^F‐FDG uptake (18), we explored the potential alternative clinical contributions of this imaging modality. Our outcomes encourage heightened consideration of cancer-associated weight loss in NSCLC patient management and substantiate prospective clinical trials to further validate the effectiveness of ^18^F‐FDG PET analysis within the workup of cancer-associated weight loss and cachexia.

## Data Availability Statement

The original contributions presented in the study are included in the article/**Supplementary Material**, further inquiries can be directed to the corresponding authors.

## Author Contributions

SO BG RI PI designed the study. DX, BG, and SO contributed to and organized the database. SO and BG performed statistical analyses. BG and SO wrote sections of the manuscript. All authors contributed to manuscript revision, read, and approved the submitted version.

## Funding

This work was supported by National Institutes of Health [P30 CA142543]; Burroughs Wellcome Fund Career Awards for Medical Scientists [1019692]; American Gastroenterological Association Scholar Award [2019AGARSA3]; American Cancer Society grant [133889-RSG-19-195-01-TBE]; Cancer Prevention and Research Institute of Texas [RP200170]; and V Foundation Scholar Award [V2019-014].

## Conflict of Interest

The authors declare that the research was conducted in the absence of any commercial or financial relationships that could be construed as a potential conflict of interest.

## Publisher’s Note

All claims expressed in this article are solely those of the authors and do not necessarily represent those of their affiliated organizations, or those of the publisher, the editors and the reviewers. Any product that may be evaluated in this article, or claim that may be made by its manufacturer, is not guaranteed or endorsed by the publisher.
