# Supplementary material for: Primary Tumor Fluorine‐18 Fluorodeoxydglucose (18F‐FDG) Is Associated With Cancer-Associated Weight Loss in Non-Small Cell Lung Cancer (NSCLC) and Portends Worse Survival
Source: Front Oncol. 2022 Jun 24;12:900712. doi: 10.3389/fonc.2022.900712 (PMC9263563; doi:10.3389/fonc.2022.900712)

Supplementary Figure 1A-D: Example of Corrected Tumor PET and L3-Level CT Images at Diagnosis for Representative Patients from Low SUV_{Max} No WL (A-B) and High SUV_{Max} WL (C-D) Groups. Erector Spinae (Green) and Psoas Muscle (Red) Groups Indicated on CT Images for Estimate of Cancer-Associated Sarcopenia.

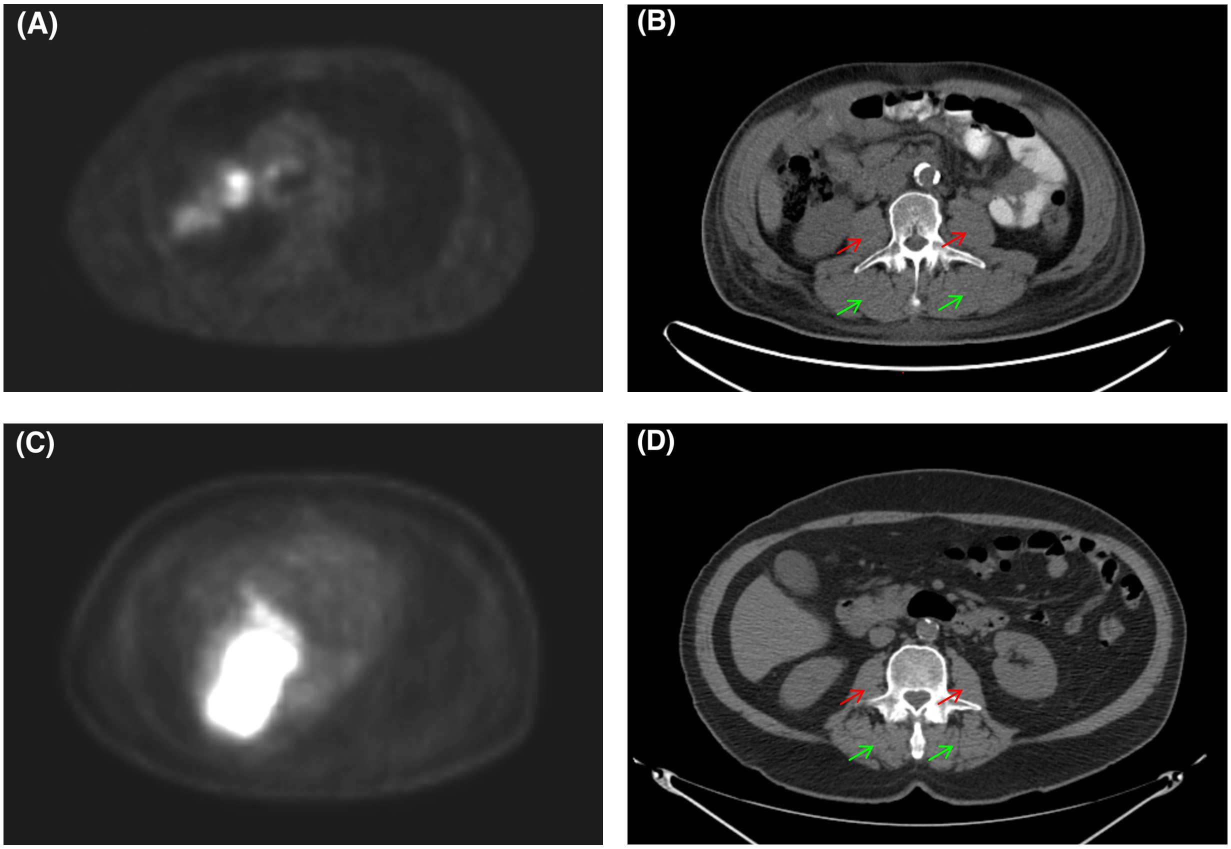

Supplementary Table 1: Multivariate Logistic Regression Evaluating Relationship of Tumor SUV_{Max} with WL Incidence at Diagnosis Including Tumor Grade as a Covariate

Patient or Tumor Factor	Odds Ratio (95% CI)	P-value
**Female Sex**	0.84 (0.493, 1.433)	*0.5227*
**Age at Diagnosis**	1.021 (0.993, 1.049)	*0.1407*
**Race**		
Non-Hispanic Caucasian	Reference	***0.0267***
Black	2.379 (1.319, 4.291)	***0.0040***
Hispanic	2.331 (0.761, 7.138)	*0.1384*
Asian or Pacific Islander	1.372 (0.215, 8.771)	*0.7384*
**Alcohol History**		
None	Reference	*0.5154*
Prior Use	1.65 (0.695, 3.916)	*0.2561*
Current Use	1.058 (0.606, 1.844)	*0.8437*
**Tobacco History**		
None	Reference	*0.6935*
Prior Use	0.825 (0.316, 2.152)	*0.6938*
Current Use	1.047 (0.393, 2.792)	*0.9261*
**Charlson Comorbidity Index**		
0	Reference	*0.2385*
1	0.521 (0.254, 1.070)	*0.0756*
2	0.568 (0.262, 1.232)	*0.1520*
3+	0.847 (0.390, 1.839)	*0.6744*
**Histology**		
Squamous	Reference	*0.1182*
Adenocarcinoma	0.555 (0.303, 1.016)	*0.0562*
Other or Unknown	1.000 (0.442, 2.263)	*0.9996*
**Stage**		
1	Reference	*0.2553*
2	0.468 (0.176, 1.244)	*0.1282*
3	0.876 (0.434, 1.767)	*0.7120*
4	1.235 (0.577, 2.641)	*0.5866*
**Grade**		
1	Reference	*0.3166*
2	1.348 (0.279, 6.510)	*0.7101*
3	2.218 (0.444, 11.082)	*0.3319*
4	1.203 (0.102, 14.187)	*0.8833*
**Tumor SUV_{Max}**	1.050 (1.011, 1.091)	***0.0110***

Supplementary Table 2: Broad Survival Comparisons

Patient Group	Median Survival Time in Months (95% CI)	P-value
**WL at Diagnosis**		***<0.0001***
No	28 (22.756, 33.244)	
Yes	16 (12.575, 19.425)	
**SUV_{Max} Cutpoint by Optimal Survival**		***<0.0001***
Low SUV	56 (42.655, 69.345)	
High SUV	19 (16.495, 21.506)	
**SUV_{Max} Cutpoint by Cohort Median**		***0.0020***
Low SUV	33 (23.288, 42.712)	
High SUV	19 (16.152, 21.848)	
**SUV_{Max} Cutpoint by Stage Median**		***0.0486***
Low SUV	28 (19.484, 36.516)	
High SUV	21 (17.129, 24.871)	

Supplementary Table 3: Log-Rank Survival Results for Survival-Optimized SUV Cutpoint

Patient Group	Median Survival Time in Months (95% CI)	Groups Compared	P-value
**Stage 1**		WL Constant	
No WL, Low SUV	96 (77.38, 114.62)	No WL: High SUV vs Low SUV	***0.0364***
No WL, High SUV	64 (32.646, 95.354)	WL: High vs Low SUV	***0.0286***
WL, Low SUV	65 (,)	SUV Constant	
WL, High SUV	30 (1.561, 58.439)	Low SUV: No WL vs WL	*0.6777*
Total	73 (53.507, 92.493)	High SUV: No WL vs WL	*0.0559*
**Stage 2**		WL Constant	
No WL, Low SUV	63 (,)	No WL: High SUV vs Low SUV	*0.1498*
No WL, High SUV	35 (2.807, 67.193)	WL: High vs Low SUV	*0.9516*
WL, Low SUV	17 (,)	SUV Constant	
WL, High SUV	44 (,)	Low SUV: No WL vs WL	*0.9683*
Total	51 (31.811, 70.189)	High SUV: No WL vs WL	*0.2835*
**Stage 3**		WL Constant	
No WL, Low SUV	23 (0, 47.58)	No WL: High SUV vs Low SUV	*0.5958*
No WL, High SUV	25 (19.454, 30.546)	WL: High vs Low SUV	*0.7155*
WL, Low SUV	13 (10.078, 15.922)	SUV Constant	
WL, High SUV	16 (10.726, 21.274)	Low SUV: No WL vs WL	*0.1704*
Total	20 (16.982, 23.018)	High SUV: No WL vs WL	***0.0201***
**Stage 4**		WL Constant	
No WL, Low SUV	17 (8.843, 25.157)	No WL: High SUV vs Low SUV	*0.2492*
No WL, High SUV	12 (8.45, 15.5)	WL: High vs Low SUV	*0.3559*
WL, Low SUV	26 (0, 69.626)	SUV Constant	
WL, High SUV	11 (9.165, 12.835)	Low SUV: No WL vs WL	*0.9043*
Total	13 (10.872, 15.128)	High SUV: No WL vs WL	*0.2089*

Supplementary Figure 2A-D: Stage Stratified Survival for WL and SUV_{Max} groups (Survival-Optimized Cutpoint)

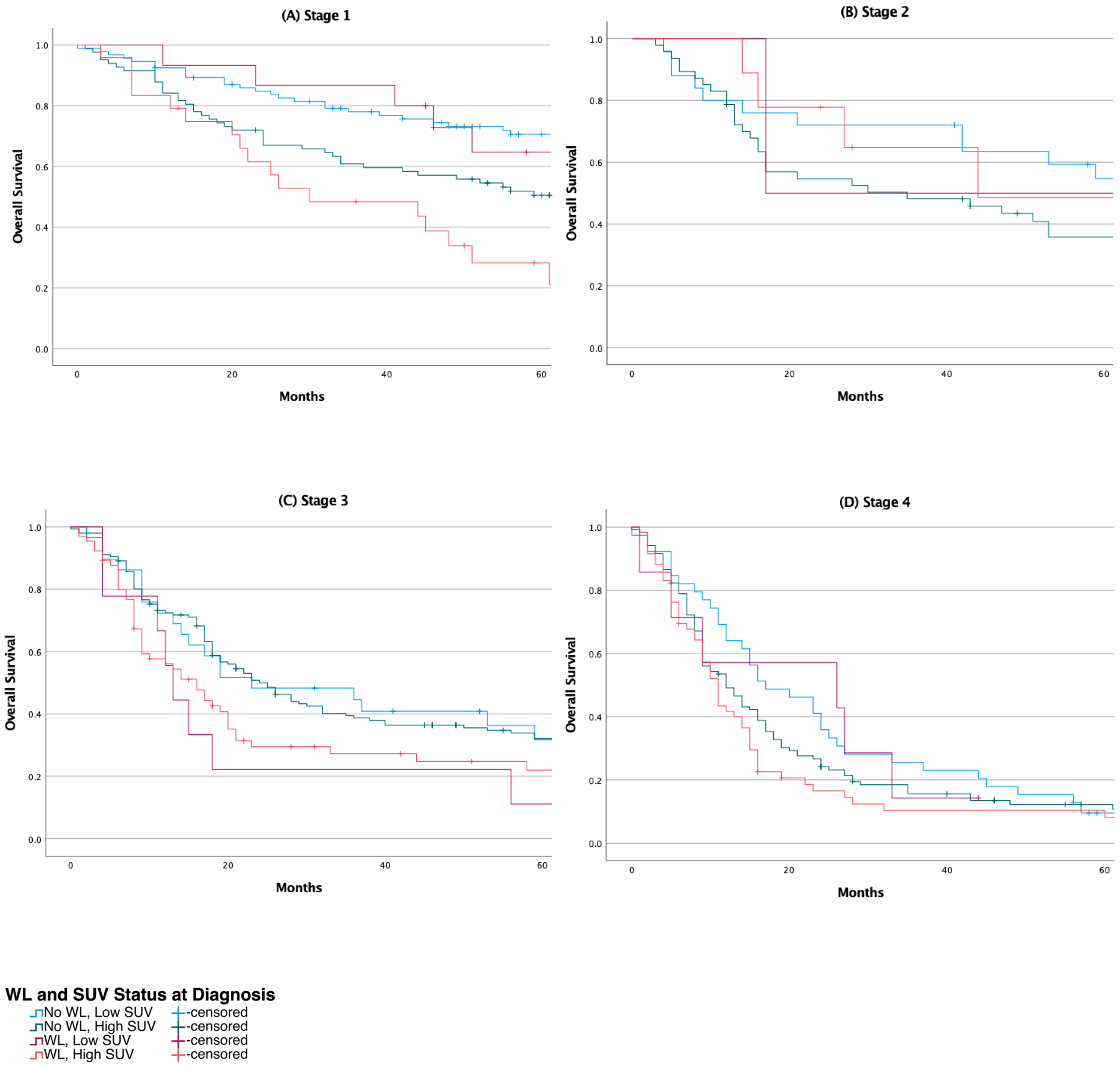

Supplementary Table 5: Log-Rank Survival Results by Total Cohort Median SUV Cutpoint

Patient Group	Median Survival Time in Months (95% CI)	Groups Compared	P-value
**Stage 1**		WL Constant	
No WL, Low SUV	95 (71.396, 118.604)	No WL: High SUV vs Low SUV	*0.9915*
No WL, High SUV	86 (41.364, 130.636)	WL: High vs Low SUV	***0.0175***
WL, Low SUV	63 (41.804, 84.196)	SUV Constant	
WL, High SUV	25 (, 53.053)	Low SUV: No WL vs WL	*0.6328*
Total	73 (53.507, 92.493)	High SUV: No WL vs WL	***0.0042***
**Stage 2**		WL Constant	
No WL, Low SUV	47 (14.301, 79.699)	No WL: High SUV vs Low SUV	*0.6401*
No WL, High SUV	51 (15.723, 86.277)	WL: High vs Low SUV	*0.0614*
WL, Low SUV	16 (13.060, 18.940)	SUV Constant	
WL, High SUV	(,)	Low SUV: No WL vs WL	*0.5978*
Total	51 (31.811, 70.189)	High SUV: No WL vs WL	*0.2723*
**Stage 3**		WL Constant	
No WL, Low SUV	26 (10.031, 41.969)	No WL: High SUV vs Low SUV	*0.8146*
No WL, High SUV	23 (15.924, 30.076)	WL: High vs Low SUV	*0.5097*
WL, Low SUV	13 (8.617, 17.383)	SUV Constant	
WL, High SUV	16 (9.392, 22.608)	Low SUV: No WL vs WL	***0.0256***
Total	20 (16.982, 23.018)	High SUV: No WL vs WL	*0.0845*
**Stage 4**		WL Constant	
No WL, Low SUV	16 (12.464, 19.536)	No WL: High SUV vs Low SUV	*0.2296*
No WL, High SUV	12 (9.027, 14.973)	WL: High vs Low SUV	*0.0565*
WL, Low SUV	15 (10.305, 19.696)	SUV Constant	
WL, High SUV	9 (6.656, 11.344)	Low SUV: No WL vs WL	*0.9395*
Total	13 (10.872, 15.128)	High SUV: No WL vs WL	*0.1464*

Supplementary Figure 3A-D: Stage Stratified Survival for WL and SUV_{Max} Groups (Total Cohort Median Cutpoint of 10.60)

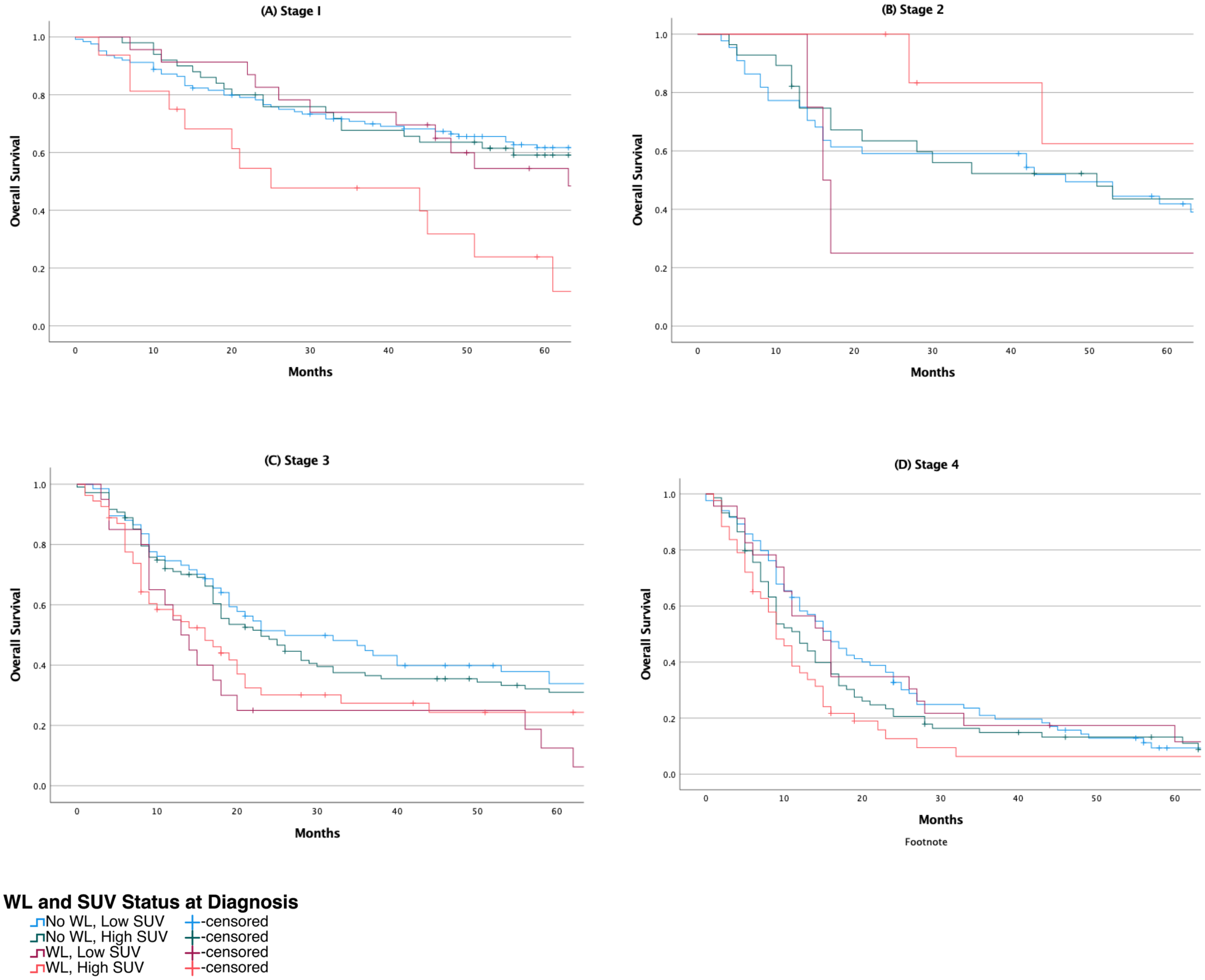

Supplementary Table 4: Log-Rank Survival Results for by Stage-Specific Median SUV Cutpoints

Patient Group	Median Survival Time in Months (95% CI)	Groups Compared	P-value
**Stage 1**		WL Constant	
No WL, Low SUV	95 (71.396, 118.604)	No WL: High SUV vs Low SUV	***0.0364***
No WL, High SUV	86 (41.364, 130.636)	WL: High vs Low SUV	***0.0286***
WL, Low SUV	63 (41.804, 84.196)	SUV Constant	
WL, High SUV	25 (0, 53.053)	Low SUV: No WL vs WL	*0.6777*
Total	73 (53.507, 92.493)	High SUV: No WL vs WL	*0.0559*
**Stage 2**		WL Constant	
No WL, Low SUV	47 (14.301, 79.699)	No WL: High SUV vs Low SUV	*0.9179*
No WL, High SUV	51 (15.723, 86.277)	WL: High vs Low SUV	*0.0614*
WL, Low SUV	16 (13.060, 18.940)	SUV Constant	
WL, High SUV	(,)	Low SUV: No WL vs WL	*0.5350*
Total	51 (31.811, 70.189)	High SUV: No WL vs WL	*0.2261*
**Stage 3**		WL Constant	
No WL, Low SUV	26 (10.031, 41.969)	No WL: High SUV vs Low SUV	*0.8184*
No WL, High SUV	23 (15.924, 30.076)	WL: High vs Low SUV	*0.9047*
WL, Low SUV	13 (8.617, 17.383)	SUV Constant	
WL, High SUV	16 (9.392, 22.608)	Low SUV: No WL vs WL	***0.0476***
Total	20 (16.982, 23.018)	High SUV: No WL vs WL	***0.0416***
**Stage 4**		WL Constant	
No WL, Low SUV	16 (12.464, 19.536)	No WL: High SUV vs Low SUV	*0.4439*
No WL, High SUV	12 (9.027, 14.973)	WL: High vs Low SUV	*0.0565*
WL, Low SUV	15 (10.305, 19.696)	SUV Constant	
WL, High SUV	9 (6.656, 11.344)	Low SUV: No WL vs WL	*0.8285*
Total	13 (10.872, 15.128)	High SUV: No WL vs WL	*0.1129*

Supplementary Figure 4A-D: Stage Stratified Survival for WL and SUV_{Max} Groups (Cutpoint by Stage-Specific Median)

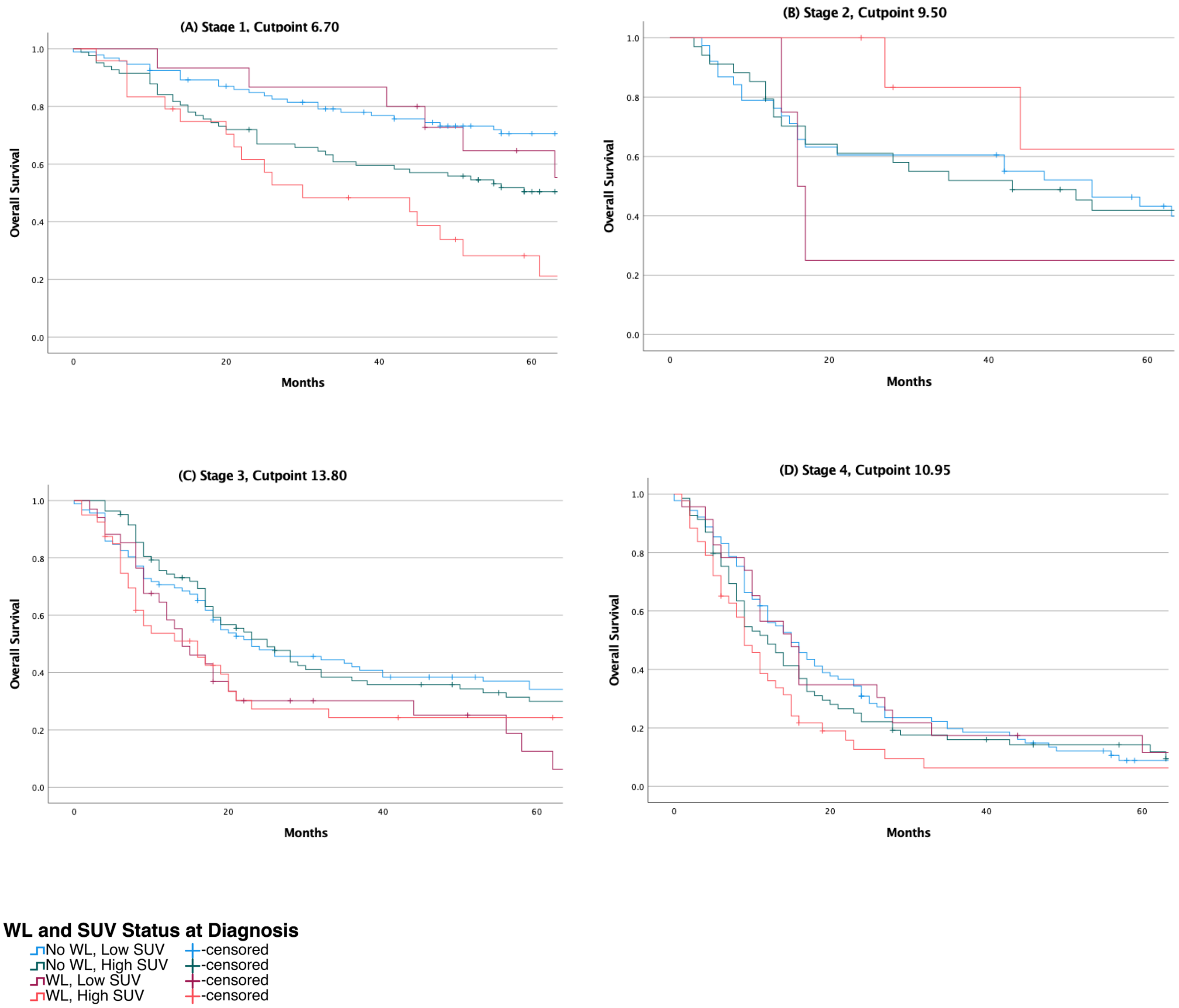

Supplement: Supplementary file 1 [file DataSheet_1.pdf]
